# Integrative Bioinformatic Analysis Identifies Key Genes Driving Breast Cancer Brain Metastasis

**DOI:** 10.3390/diagnostics16081149

**Published:** 2026-04-13

**Authors:** Wei-Yi Ting, Yueh-Hsun Lu, Che-Ming Lin

**Affiliations:** 1 Department of Radiology, Shuang-Ho Hospital, Taipei Medical University, New Taipei City 23561, Taiwan; twyalen@gmail.com (W.-Y.T.); 20001@s.tmu.edu.tw (Y.-H.L.); 2 Department of Radiology, School of Medicine, College of Medicine, Taipei Medical University, New Taipei City 23561, Taiwan

**Keywords:** breast cancer, brain metastasis, bioinformatics, biomarker, cell cycle, metabolism

## Abstract

**Background/Objectives:** Brain metastasis (BM) represents a significant clinical challenge in advanced breast cancer, yet the molecular mechanisms driving breast cancer brain metastasis (BCBM) remain incompletely characterized. This study aims to identify key molecular pathways and hub genes specifically associated with BCBM through comprehensive bioinformatic analyses. **Methods:** Gene Set Enrichment Analysis (GSEA), differential gene expression analysis, and weighted gene co-expression network analysis (WGCNA) were performed using two independent GEO datasets (GSE191230 and GSE43837). Protein–protein interaction (PPI) networks were constructed to visualize functional interconnections among dysregulated genes. Survival analyses were conducted using the Kaplan–Meier Plotter database to evaluate the prognostic significance of identified hub genes. **Results:** GSEA revealed significant upregulation of metabolic pathways (mTORC1 signaling, glycolysis, oxidative phosphorylation) and downregulation of immune-related pathways in BCBM compared to primary tumors. Integrative analysis identified 34 consistently dysregulated genes across datasets, from which 12 hub genes were validated. Among these, RRM2, CDCA8, CCNB1, LMNB2, FANCI, NCAPH, YWHAZ, and ESPL1 demonstrated brain-specific over-expression compared to other metastatic sites. Functional enrichment analysis highlighted cell cycle dysregulation as a critical mechanism in BCBM, and all hub genes showed significant association with poor prognosis in breast cancer patients. **Conclusions:** This study identifies a unique molecular profile of BCBM characterized by cell cycle dysregulation, metabolic reprogramming, and immune microenvironment alterations. The brain-specific expression patterns of these hub genes represent potential biomarkers for BCBM risk assessment and novel therapeutic targets, providing a basis for precision medicine development.

## 1. Introduction

Breast cancer is one of the most common malignancies worldwide. In 2020, approximately 2.3 million women were diagnosed with breast cancer, and there were 685,000 deaths from the disease [1,2]. It is also the leading cause of cancer death among women globally [3]. Due to advances in early detection, surgery, and treatment methods, breast cancer survival rates have increased [4]. However, when the cancer spreads to other parts of the body, it remains the main cause of death, and the 5-year survival rate becomes significantly reduced once distant metastasis occurs [5,6]. The most common sites of distant recurrence for breast cancer are the brain, bones, lung, and liver [6].

Brain metastasis (BM) is a serious issue in advanced breast cancer, which impacts approximately 10–30% of patients with metastatic disease [6]. The incidence of brain metastasis has been increasing in recent years. This rise has been attributed to the development of better imaging and screening techniques, as well as the advancement of more effective systemic therapies [7]. BM is associated with severe neurological complications, reduced quality of life, and poor prognosis. Median survival varies from 2 to 20 months, depending on prognostic factors and treatment approaches [8,9].

The tendency of BM differs among breast cancer types [10]. Human epidermal growth factor receptor 2 (HER2)-positive and triple-negative breast cancer (TNBC) show higher rates of BM compared to luminal types [10]. This subtype-specific tropism suggests distinct molecular mechanisms driving brain colonization in different breast cancer subtypes [11]. Previous studies have identified several molecular pathways potentially involved in breast cancer brain metastasis (BCBM). HER2 over-expression has been associated with increased blood–brain barrier (BBB) permeability and enhanced brain colonization through activation of downstream signaling pathways [12]. Similarly, genes involved in migration (HBEGF, CXCR4) [13,14,15], protein degradation (ADAM8, Cathepsin S) [16,17], immune modulation (GBP1, COX-2) [13,18], and angiogenesis (Angiopoietin-2) [19] have been implicated in brain-specific metastasis. Furthermore, metabolic adaptations allowing cancer cells to utilize alternative energy sources such as glutamine in the nutrient-restrictive brain microenvironment have been reported [20,21].

Currently, there are limited prognostic biomarkers available to predict the risk of BM in breast cancer patients. Clinical parameters such as young age, tumor size, nodal status, and hormone receptor negativity have shown some association with BM risk [22]. Several gene expression signatures have been proposed; Lee et al. (2016) identified a panel of genes including CXCL12, MMP-2, MMP-11, VCAM1, and MME that showed prognostic significance in BM patients [23]. However, these signatures have not been widely validated or implemented in clinical practice. Additionally, Iwamoto et al. (2019) reported that immune-related gene signatures exhibited significantly lower expression in BM compared to primary breast cancers, suggesting that the immune-desert microenvironment of BM may contribute to treatment resistance [24]. Their study also identified VEGFA and DNMT3A as potential therapeutic targets specifically upregulated in BM [24]. Furthermore, circulating biomarkers such as circulating tumor cells (CTCs) expressing EGFR, heparanase (HPSE), and NOTCH1 have shown potential for predicting BM [25]. However, these biomarkers lack specificity and provide limited insights into the underlying molecular mechanisms governing the brain metastatic process.

Despite advances in our understanding of BCBM, several critical knowledge gaps remain. First, the molecular determinants specifically leading to brain tropism, as opposed to metastasis to other organs, are still incompletely understood. While certain genes and pathways have been implicated in BM, a comprehensive characterization of the molecular networks distinguishing brain-specific metastasis from metastasis to other sites is lacking. Second, the identification of robust and clinically applicable biomarkers for predicting BM risk remains an unmet need. Finding patients who are at high risk for brain metastasis early may allow for preventive interventions. Furthermore, there is limited understanding of how these BM-associated genes interact within functional networks and biological pathways. Most previous studies have focused on individual genes or limited gene sets without considering broader pathway interactions and co-expression patterns. Using a systematic approach that combines differential gene expression analysis with gene co-expression network analysis can provide deeper insights into the complex molecular activities of brain metastatic breast cancer.

Given these knowledge gaps, the present study aims to comprehensively characterize the molecular landscape of BCBM through integrative bioinformatic approaches. We aim to advance our understanding of the molecular basis of BCBM, identify novel biomarkers for risk stratification, and uncover potential therapeutic targets for this devastating complication of advanced breast cancer. The integrative bioinformatic approach employed in this study provides a systems-level perspective on the complex biological processes that drive brain-specific metastasis, potentially informing the development of precision medicine strategies to improve outcomes for patients at risk of brain metastatic disease.

## 2. Materials and Methods

### 2.1. Data Sources

The datasets used in this study—GSE191230, GSE43837, and GSE14018—were obtained from the Gene Expression Omnibus (GEO) database. The detailed download sources are listed in Supplementary Table S1. GSE191230 comprised 13 primary breast tumor samples and 7 brain metastasis samples. GSE43837 comprised 19 primary breast cancer samples and 19 brain metastasis samples. As the two datasets were derived from different technological platforms (RNA-seq and microarray, respectively), DEG analyses were performed independently within each dataset, and only concordantly upregulated genes were retained via Venn diagram intersection.

### 2.2. Gene Set Enrichment Analysis (GSEA)

GSEA was conducted via the Broad Institute platform, employing the default weighted Signal2Noise metric. For the GSE191230 dataset, gene expression profiles were ranked according to the expression levels of selected candidate genes. Samples were stratified into high- and low-expression groups based on whether their expression levels were above or below the median across all candidate genes. Gene sets were deemed significantly enriched when the absolute value of the normalized enrichment score (|NES|) exceeded 1, the false discovery rate (FDR) was below 0.25, and the nominal *p*-value was less than 0.05, as determined from 1000 random permutations.

### 2.3. Construction of Weighted Gene Co-Expression Network Analysis (WGCNA)

After downloading GSE14018, probe IDs were converted to gene symbols using gene annotation files. In cases where multiple probe IDs corresponded to the same gene, the probe with the highest expression value was retained. Gene expression data from the GSE14018 matrix were ranked based on variance, and the top 50% of genes were selected for WGCNA using the R package WGCNA (1.74). The adjacency matrix was then transformed into a topological overlap matrix (TOM), and genes were clustered into modules based on TOM-based dissimilarity. A soft-thresholding power of 5, a cut height of 0.25, and a minimum module size of 30 were used to identify the gene modules.

### 2.4. Identification of Differentially Expressed Genes (DEGs) and Hub Gene Selection

The NCBI GEO database was utilized for the analysis of two datasets: GSE191230 and GSE43837. The raw data were processed using the GEO2R online tool (https://www.ncbi.nlm.nih.gov/geo/geo2r/ accessed on 10 January 2025), retaining only genes with a *p* < 0.05, a log2 fold-change > 0.5, and a higher expression in brain metastasis samples relative to primary breast tumors. The overlapping DEGs between both datasets were then compared with genes in the turquoise module identified by WGCNA using Venn software (2.1.0), yielding 34 consistently upregulated genes representing a high-confidence core set of brain metastasis-associated genes. To further refine this gene set, each candidate gene was required to demonstrate statistically significant upregulation in breast cancer tumor samples (num(T) = 1085) relative to normal breast tissue (num(*N*) = 291), as assessed by box plot analysis using the BRCA (Breast Invasive Carcinoma) dataset (*p* < 0.05). This criterion yielded the final 12 hub genes, namely RRM2, NETO2, ESPL1, CDCA8, CCNB1, LMNB2, SAC3D1, NCAPH, MCM2, YWHAZ, FANCI, and FOXRED2, which demonstrated consistent and statistically significant upregulation in both brain metastasis relative to primary breast tumors and tumor tissue relative to normal breast tissue (Table S3).

### 2.5. Function Enrichment Analysis

The turquoise module, which exhibited a strong correlation with brain metastasis, underwent Gene Ontology (GO) and Kyoto Encyclopedia of Genes and Genomes (KEGG) enrichment analyses using Metascape, an online gene functional classification tool (https://metascape.org/gp/index.html#/citations/ accessed on 10 January 2025). Enrichment was confirmed when *p* < 0.05. Enrichment bar graphs and networks were generated to visualize the results.

### 2.6. Kaplan–Meier Survival Analysis

The prognostic values of distant metastasis-free survival (DMFS) for brain metastatic breast cancer were evaluated using available data and tools from the Kaplan–Meier Plotter website (http://kmplot.com/analysis/index.php?p=background accessed on 10 January 2025). The data were obtained from the GEO database, and the candidate genes (RRM2, CDCA8, CCNB1, LMNB2, FANCI, NCAPH, YWHAZ, NETO2, MCM2, and ESPL1) were used as input. Kaplan–Meier plots were drawn, log-rank tests were performed to analyze differences between risk groups, and hazard ratios were estimated.

### 2.7. Statistical Analysis

Survival curves were generated using GraphPad Prism V9.5.1 (GraphPad Software, Inc., San Diego, CA, USA) through the Kaplan–Meier method. The differences between the gene expression groups were assessed using the log-rank test. The correlation coefficients were calculated using the Pearson correlation test, created using GraphPad Prism. Student’s *t*-test was utilized for statistical analysis comparing gene expression between the brain and other sites. A *p*-value less than 0.05 was considered statistically significant.

## 3. Results

### 3.1. Enrichment of Metabolic and Inflammatory Signaling Pathways in Brain Metastatic Breast Cancer

**Figure 1:**
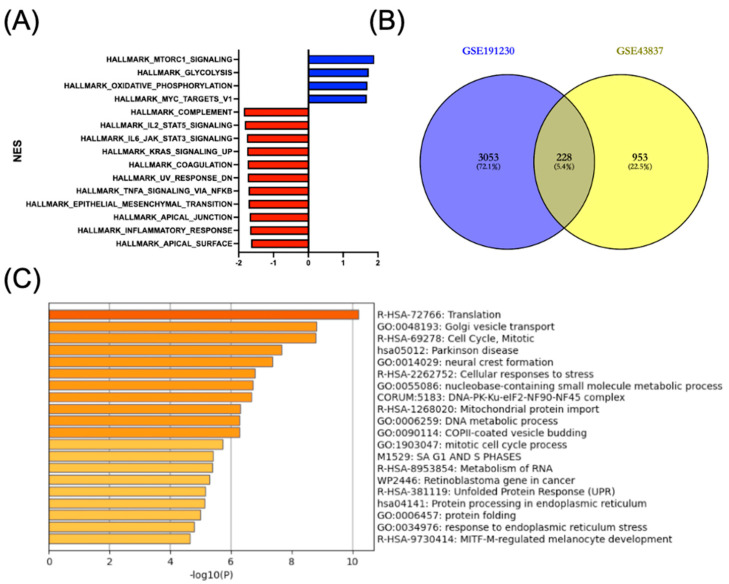
Gene Set Enrichment Analysis and differential gene expression in brain metastatic breast cancer. (**A**) GSEA of hallmark gene sets comparing brain metastatic breast cancer to primary breast tumors. Normalized enrichment scores are shown on the *x*-axis, with positive scores (blue bars) indicating upregulation and negative scores (red bars) indicating downregulation in brain metastases. (**B**) Venn diagram showing the overlap of DEGs between two independent datasets (GSE191230 and GSE43837). Numbers indicate unique and shared DEGs with their respective percentages. (**C**) Functional enrichment analysis of DEGs. The bar chart displays significantly enriched biological processes, pathways, and complexes with their corresponding −log10 (*p*-value) on the *x*-axis.

To identify key molecular mechanisms and biological processes driving breast cancer metastasis to the brain, we performed GSEA and differential gene expression analysis. GSEA revealed distinct patterns of pathway enrichment in BM samples compared to primary breast tumors (Figure 1A). Notably, metabolic pathways including mTORC1 signaling, glycolysis, and oxidative phosphorylation were significantly upregulated (normalized enrichment score > 0, blue bars), suggesting metabolic reprogramming as an important characteristic of brain metastatic cells. MYC targets were also enriched, consistent with the known role of MYC in promoting cancer cell proliferation and metabolic adaptation [26].

In contrast, multiple inflammatory and immune-related pathways showed significant downregulation (normalized enrichment score < 0, red bars), including the complement cascade, IL2-STAT5 signaling, IL6-JAK-STAT3 signaling, KRAS signaling, coagulation, UV response, and TNFα signaling via NF-κB (Figure 1A) pathways. Furthermore, pathways related to cellular structure and epithelial characteristics such as epithelial–mesenchymal transition, the apical junction, inflammatory response, and the apical surface were also downregulated. These findings suggest that brain metastatic breast cancer cells may escape immune surveillance while undergoing metabolic adaptation to the brain microenvironment.

Transcriptomic comparison between BM and primary tumors identified a significant number of DEGs. The Venn diagram analysis of DEGs from two independent datasets (GSE191230 and GSE43837) demonstrated 3053 unique DEGs in GSE191230 (72.1%) and 953 unique DEGs in GSE43837 (22.5%), with 228 common DEGs (5.4%) between both datasets (Figure 1B; Table S2). This overlap represents a core set of consistently altered genes in brain metastatic breast cancer across different patient cohorts. The overlap of 228 common DEGs between GSE191230 and GSE43837 represents a statistically significant enrichment beyond chance expectation (hypergeometric test, *p* = 3.61 × 10^−3^, fold enrichment = 1.18×, background *N* = 20,000 genes), reinforcing the biological reproducibility and cross-cohort reliability of this core DEG set as a foundation for subsequent integrative analysis.

Functional enrichment analysis of the DEGs identified several significantly enriched biological processes and pathways (Figure 1C). The most significantly enriched terms included translation, Golgi vesicle transport, cell cycle regulation, Parkinson disease-related genes, neural crest formation, and cellular responses to stress. Additional enriched processes included nucleobase-containing small-molecule metabolic processes, the DNA-PK-Ku-eIF2-NF90-NF45 complex, mitochondrial protein import, DNA metabolic processes, COPII-coated vesicle budding, and mitotic cell cycle regulation. Cancer-related and stress response pathways including retinoblastoma gene signaling, RNA metabolism, unfolded protein response, protein processing in the endoplasmic reticulum, protein folding, and response to endoplasmic reticulum stress were also significantly enriched.

### 3.2. Gene Co-Expression Module Identification in Metastatic Breast Cancer

**Figure 2:**
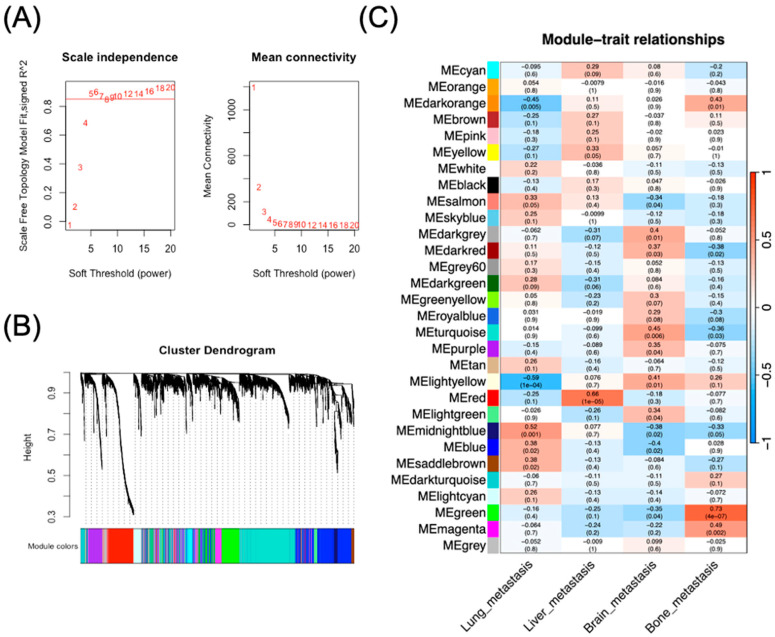
Weighted gene co-expression network analysis of breast cancer metastasis. (**A**) Determination of soft-thresholding power for WGCNA. Left panel: Scale-free topology model fit (*y*-axis) as a function of soft-thresholding power (*x*-axis). The red line indicates R^2^ = 0.8, with power = 5 selected as the optimal threshold. Right panel: Mean connectivity (*y*-axis) as a function of soft-thresholding power (*x*-axis), showing the relationship between network connectivity and increasing power values. (**B**) Cluster dendrogram showing gene modules identified by WGCNA. The hierarchical clustering tree was constructed based on TOM. Each color in the bar below the dendrogram represents a distinct gene module. (**C**) Heatmap of module–trait relationships. Rows represent module eigengenes and columns represent four metastatic sites (lung, liver, brain, and bone metastasis). Each cell contains the correlation coefficient (top number) and corresponding *p*-value (bottom number in parentheses). Red indicates positive correlation, blue indicates negative correlation, and white indicates no correlation. Color intensity corresponds to correlation strength.

To gain deeper insights into the transcriptional networks and gene modules driving organ-specific metastasis in breast cancer, particularly BM, we performed WGCNA. This approach enables identification of clusters of highly interconnected genes and their associations with specific clinical traits or metastatic sites. The scale independence analysis showed that a soft-thresholding power of 5 achieved a high scale-free topology model fit (R^2^ > 0.8) while maintaining sufficient mean connectivity (Figure 2A). This power parameter was subsequently used for network construction to identify gene modules with coordinated expression patterns. Hierarchical clustering resulted in the identification of 29 distinct gene modules, as illustrated in the cluster dendrogram (Figure 2B).

To assess the biological significance of these modules in the context of organ-specific metastasis, we correlated module eigengenes (ME) with four distinct metastatic phenotypes: lung metastasis, liver metastasis, brain metastasis, and bone metastasis (Figure 2C). The heatmap of module–trait relationships revealed several modules with significant associations to specific metastatic sites. Notably, the MEturquoise module demonstrated the strongest positive correlation specifically with brain metastasis (r = 0.45, *p* = 0.006), highlighting potential brain-specific metastatic drivers. By comparison, the MEmidnightblue module showed a statistically significant negative correlation with brain metastasis (r = −0.38, *p* = 0.02), and the MEred module showed a weak, non-significant negative correlation (r = −0.18, *p* = 0.3), confirming that MEturquoise was the most robustly and positively associated module to brain-specific metastatic progression and it was therefore selected for downstream analysis. Conversely, the MEblue module showed the strongest negative correlation with brain metastasis (r = −0.4, *p* < 0.05), suggesting its potential suppressive roles in brain metastatic progression. The identification of these site-specific modules provides a foundation for further exploration of the molecular determinants governing organ-specific metastasis, particularly the distinctive mechanisms leading to brain metastasis in breast cancer.

### 3.3. Pathway Analysis of Brain Metastasis Gene Signatures

After identifying differential gene expression patterns and co-expression modules associated with BM, we sought to characterize the functional landscape of these BM-associated genes through detailed pathway enrichment analysis and protein–protein interaction (PPI) network mapping. This approach allows for the identification of key biological processes and molecular interactions that may lead to brain-specific metastasis in breast cancer.

**Figure 3:**
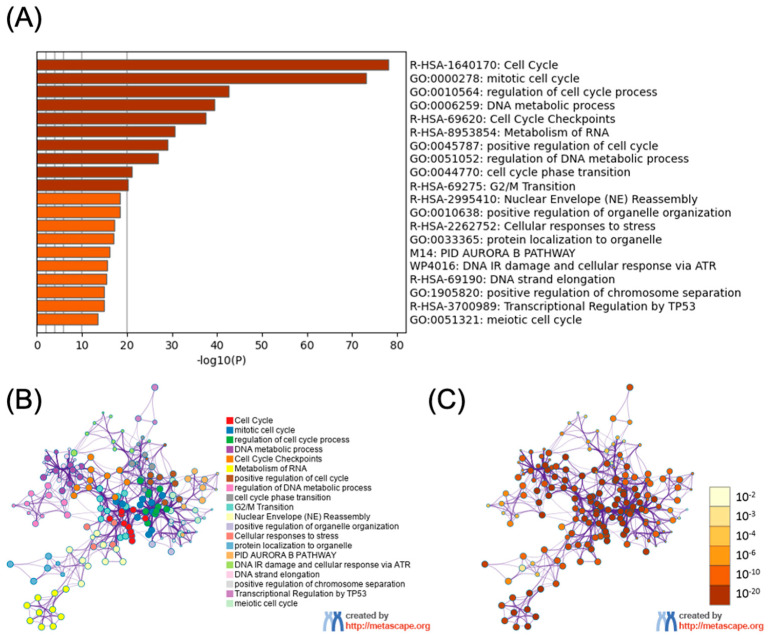
Functional enrichment and PPI network analysis of brain metastasis-associated genes. (**A**) Functional enrichment analysis of genes associated with BCBM. The bar chart displays significantly enriched biological processes, pathways, and cellular functions, with their corresponding statistical significance expressed as −log10(P) values on the *x*-axis. (**B**) PPI network of BM-associated genes. Nodes represent proteins, and edges represent known or predicted interactions between proteins. Nodes are color-coded according to the functional categories identified in panel A, illustrating the clustering of proteins involved in related biological processes. (**C**) Significance-weighted PPI network. The same network structure as in panel B but with nodes colored according to their statistical significance of association with BM, ranging from yellow (less significant, 10^−2^) to dark brown (highly significant, 10^−20^) as indicated in the color scale.

Functional enrichment analysis of genes significantly associated with BM is predominantly related to cell cycle-related processes (Figure 3A). The most significantly enriched pathway was “cell cycle” (R-HSA-1640170), followed by various cell cycle regulatory processes including “mitotic cell cycle” (GO:0000278), “regulation of cell cycle process” (GO:0010564), and “DNA metabolic process” (GO:0006259). Cell cycle checkpoints (R-HSA-69620) were also highly enriched, suggesting that dysregulation of cell cycle control may be a critical feature of brain metastatic breast cancer cells. Notably, the PID AURORA B PATHWAY (M14) was enriched, highlighting the potential importance of Aurora B kinase signaling in brain metastasis.

To visualize the interactions among these functionally enriched genes, we constructed a PPI network (Figure 3B). Cell cycle-related proteins formed the most notable clusters, with extensive interconnections suggesting coordinated regulation of cell cycle processes in brain metastasis. Further analysis of the PPI network incorporating statistical significance of each node revealed a gradient of significance levels across the network (Figure 3C). Collectively, these analyses highlight cell cycle regulation as a crucial mechanism potentially driving breast cancer metastasis to the brain, with particular emphasis on mitotic processes, DNA metabolism, and cell cycle checkpoints. These findings provide a basis for identifying novel therapeutic targets specifically for brain metastatic breast cancer.

### 3.4. Identification of Key Genes in Brain Metastatic Breast Cancer

**Figure 4:**
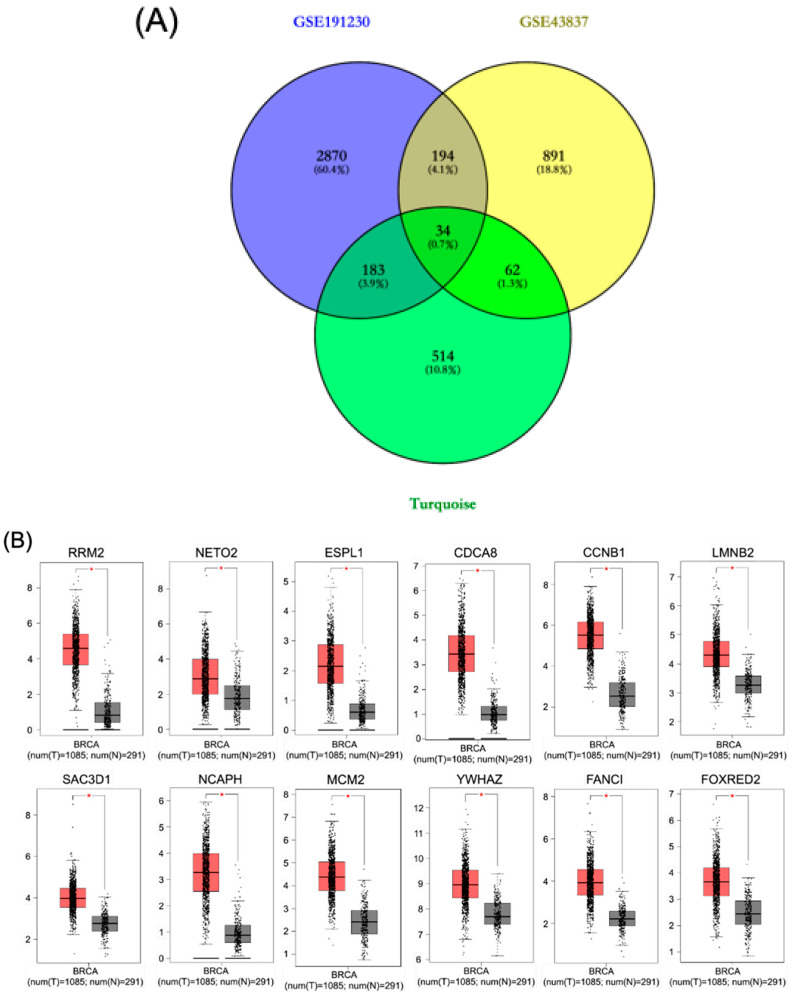
Identification and validation of consistently dysregulated genes in brain metastatic breast cancer. (**A**) Venn diagram showing the overlap of DEGs across three independent datasets: GSE191230 (blue circle), GSE43837 (yellow circle), and the turquoise module identified from WGCNA (green circle). Numbers represent gene counts in each region with their respective percentages in parentheses. (**B**) Box plot validation of expression levels for 12 selected key genes consistently upregulated in BM. Each panel represents the expression level of an individual gene (RRM2, NETO2, ESPL1, CDCA8, CCNB1, LMNB2, SAC3D1, NCAPH, MCM2, YWHAZ, FANCI, and FOXRED2) in primary breast cancer samples (gray boxes, *n* = 291) versus brain metastasis samples (red boxes, *n* = 1085). The *y*-axis represents normalized expression values, with higher values indicating increased expression. Statistical significance is indicated as follows: * *p* < 0.05.

To identify reliable gene signatures specifically associated with BM in breast cancer, we performed an integrative analysis across multiple independent datasets. This approach aimed to overcome the limitations of single-dataset analyses by identifying consistently dysregulated genes across different patient cohorts. We first conducted a comparative analysis of DEGs identified in three independent datasets: GSE191230, GSE43837, and the previously identified turquoise module from our WGCNA (Figure 4A). The Venn diagram illustrates the distribution and overlap of DEGs across these datasets. Most importantly, we identified 34 genes (0.7%) consistently present across all three datasets, representing a core set of highly reproducible brain metastasis-associated genes.

To validate the expression patterns and potential functional significance of key genes from this crucial core, we identified 12 key genes that show consistent and significant upregulation in brain metastasis samples (red boxes) compared to primary breast cancer samples (gray boxes) (Figure 4B). These hub genes include RRM2 (Ribonucleotide reductase regulatory subunit M2), NETO2 (Neuropilin and tolloid-like 2), ESPL1 (Extra spindle pole bodies like 1, Separase), CDCA8 (Cell division cycle associated 8), CCNB1 (Cyclin B1), LMNB2 (Lamin B2), SAC3D1 (SAC3 domain containing 1), NCAPH (Non-SMC condensin I complex subunit H), MCM2 (Minichromosome maintenance complex component 2), YWHAZ (Tyrosine 3-monooxygenase/Tryptophan 5-monooxygenase activation protein zeta), FANCI (FA complementation group I), and FOXRED2 (FAD-dependent oxidoreductase domain containing 2). Expression validation was performed using GEPIA (Gene Expression Profiling Interactive Analysis; http://gepia.cancer-pku.cn/ accessed on 10 January 2025) with TCGA (The Cancer Genome Atlas) BRCA (Breast Invasive Carcinoma) dataset (num(T) = 1085 tumor samples; num(N) = 291 normal breast tissue samples). Over-expression was defined uniformly across all 12 hub genes using an absolute log2 fold-change cutoff of |Log2FC| ≥ 1 and a significance threshold of *p* < 0.05. All 12 hub genes met both criteria, demonstrating statistically significant upregulation in breast cancer tumor tissue relative to normal breast tissue (Figure 4B).

The consistent upregulation of these genes across different patient cohorts strongly suggests their functional importance in brain metastasis. Particularly, many of these genes are involved in cell cycle regulation (RRM2, ESPL1, CDCA8, CCNB1, MCM2), chromosome organization (NCAPH, LMNB2), and DNA damage repair (FANCI), which aligns with our earlier pathway enrichment findings highlighting cell cycle dysregulation as a key feature of brain metastatic breast cancer. These findings collectively identify a core set of consistently dysregulated genes in brain metastatic breast cancer, which may serve as potential biomarkers and therapeutic targets for this clinically challenging disease.

### 3.5. Prognostic Significance of Key Genes in Breast Cancer Patient Survival

**Figure 5:**
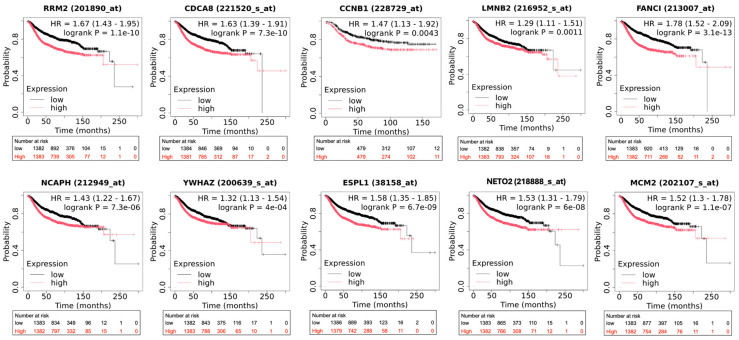
Kaplan–Meier survival analysis of key genes in breast cancer patients. Kaplan–Meier plots showing the relationship between gene expression levels and DMFS in breast cancer patients for ten hub genes: RRM2, CDCA8, CCNB1, LMNB2, FANCI, NCAPH, YWHAZ, ESPL1, NETO2, and MCM2. For each gene, patients were stratified into high-expression (red curves) and low-expression (black curves) groups based on median expression values. The *x*-axis represents time in months, and the *y*-axis represents survival probability. Each plot includes the HR with the 95% confidence interval, log-rank *p*-value, and the number of patients at risk at different time points. Gene-specific probe IDs are indicated in each plot.

After identifying and validating the consistent upregulation of key genes in brain metastatic breast cancer, we sought to determine whether these genes have clinical relevance as prognostic biomarkers. Survival analysis was performed to evaluate the relationship between the expression levels of our identified key genes and patient outcomes in breast cancer. Using the Kaplan–Meier Plotter analysis, all ten analyzed key genes demonstrated significant associations with poor prognosis, with high expression consistently correlating with reduced DMFS in breast cancer patients (Figure 5). The most significant negative prognostic effect was observed for FANCI, with a hazard ratio (HR) of 1.78, which was the highest HR among all analyzed genes. Similarly, RRM2 exhibited a strong negative prognostic effect with an HR = 1.67.

Notably, NETO2, a gene involved in neuronal function, showed a significant association with poor prognosis (HR = 1.53), suggesting its potential role in brain metastasis. Additionally, the cellular signaling regulator YWHAZ also demonstrated a significant negative prognostic effect with an HR = 1.32, highlighting the importance of altered signaling pathways in breast cancer progression. The consistent association of these key genes with poor prognosis in breast cancer patients strongly supports their potential roles as drivers of metastatic progression. Furthermore, the prognostic significance of these genes across a large patient cohort validates the clinical relevance of our bioinformatic findings and suggests that these genes may serve as valuable biomarkers for risk stratification and potential therapeutic targets.

### 3.6. Differential Expression Analysis of Key Genes in Brain Versus Other Metastatic Sites in Breast Cancer

**Figure 6:**
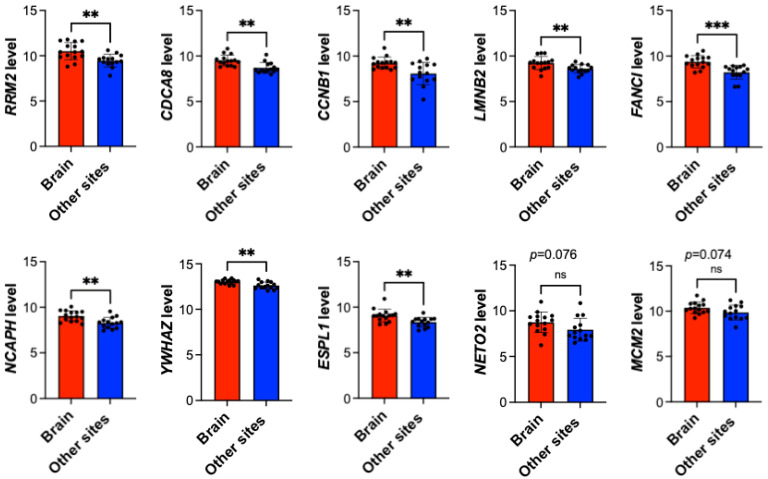
Comparison of key gene expression levels between brain metastases and other metastatic sites in breast cancer. Bar graphs comparing the expression levels of ten key genes (RRM2, CDCA8, CCNB1, LMNB2, FANCI, NCAPH, YWHAZ, ESPL1, NETO2, and MCM2) between breast cancer metastases in the brain (red bars) and other metastatic sites (blue bars). The *y*-axis represents normalized gene expression levels, and individual data points are shown as black dots. Statistical significance is indicated as follows: ** *p* < 0.01, *** *p* < 0.001, ns (not significant). Error bars represent standard deviation.

To further characterize the organ specificity of our identified key genes and determine whether their upregulation is specifically associated with BM or represents a general feature of metastatic breast cancer, we compared their expression levels between BM and metastases at other anatomical sites. This analysis aimed to identify genes with brain-specific over-expression patterns, which could serve as specific biomarkers or therapeutic targets for BM. Remarkably, eight of the ten key genes showed significantly higher expression in BM (red bars) compared to other metastatic sites (blue bars), supporting their specific association with brain tropism in metastatic breast cancer (Figure 6).

The most significant differential expression was observed for FANCI, suggesting its particularly strong association with BM (*p* < 0.001). Seven other genes showed significant upregulation in BM with *p* < 0.01. Remarkably, RRM2, CDCA8, CCNB1, LMNB2, FANCI, and NCAPH, which are involved in cell cycle regulation and DNA replication, were specifically upregulated in BM, suggesting that enhanced proliferative capacity might be a characteristic of breast cancer cells that metastasize to the brain. Interestingly, two genes, NETO2 and MCM2, did not show statistically significant differences between the brain and other metastatic sites (*p* = 0.076 and *p* = 0.074, respectively). This suggests that while these genes are upregulated in metastatic breast cancer in general, their expression is not specifically elevated in BM compared to other metastatic sites. However, it is noteworthy that both NETO2 and MCM2 showed trends toward higher expression in BM.

These findings collectively support the hypothesis that BM in breast cancer is associated with a distinct molecular profile, characterized by the upregulation of specific genes involved in cell cycle regulation, DNA replication, and cellular signaling. The brain-specific expression patterns of these key genes provide further evidence for their potential application as specific biomarkers for BM risk assessment and as targets for brain-specific therapeutic interventions in metastatic breast cancer.

## 4. Discussion

### 4.1. Major Findings and Implications

BM represents one of the most disturbing complications of advanced breast cancer due to its association with significant neurological illness and poor prognosis. Through our comprehensive bioinformatic analysis of gene expression profiles, we have identified several key molecular pathways and potential biomarkers of BCBM that may contribute to improved diagnostic and therapeutic approaches for this challenging clinical issue.

Our GSEA study demonstrated significant upregulation of metabolic pathways, including mTORC1 signaling, glycolysis, and oxidative phosphorylation in BM compared to primary breast tumors. This metabolic reprogramming potentially reflects the adaptation of cancer cells to the unique nutrient availability in the brain microenvironment. The brain is a highly energy-demanding organ that primarily uses glucose as an energy source, and our findings suggests that breast cancers that successfully colonize the brain have adapted to effectively compete for these energy sources. This is consistent with previous studies showing that metastatic cells experience metabolic adaptation to survive and proliferate in the brain microenvironment [27,28]. Targeting these altered metabolic pathways could potentially disrupt the ability of breast cancer cells to colonize the brain microenvironment.

A remarkable finding of our study was the significant downregulation of immune and inflammatory response pathways in BM. These include the complement cascade, IL2-STAT5 signaling, IL6-JAK-STAT3 signaling, and TNFα signaling via NF-κB. Our observation aligns with the findings of Iwamoto et al. (2019), who reported decreased immune-related gene signatures in BM compared to primary breast tumors [24]. The downregulation of these immune-related pathways suggests that BCBM may create an immunosuppressive microenvironment, which potentially leads to treatment resistance. In fact, studies have demonstrated this immune-desert phenotype, which might partially explain the limited efficacy of immune checkpoint inhibitors in BCBM [29,30,31]. Strategies to enhance immune cell infiltration and activation within BM could be a potential direction for further investigation.

It is important to note, however, that the observed downregulation of IL2/STAT5, IL6/JAK/STAT3, and TNFα/NF-κB signaling in BM bulk transcriptomic data may reflect, at least in part, a compositional effect rather than solely tumor-intrinsic immunosuppression. The brain is an immunologically distinct organ with restricted immune cell trafficking across the blood–brain barrier and markedly lower infiltration of peripheral lymphocytes and NK cells compared to primary breast tumors. Consequently, the reduced transcriptional signal from immune-related pathways in BM bulk tissue may partly reflect the physical absence of immune effector cells that normally produce these signals, rather than active suppression by tumor cells. Resolving these two mechanisms will require computational immune deconvolution or single-cell transcriptomic approaches, both of which are priorities for future investigation.

Our functional enrichment analysis and PPI network mapping demonstrated a noticeable dominance of cell cycle-related processes among BM-associated genes. The significant enrichment of cell cycle regulation, DNA metabolic processes, and mitotic phase transitions suggests that dysregulation of the cell proliferation mechanism is a crucial step in brain metastatic progression. This is further supported by our identification of several key genes involved in cell cycle regulation (RRM2, CDCA8, CCNB1, MCM2) and chromosome organization (NCAPH, LMNB2) that were consistently upregulated in BM. Our identification of the critical role of cell cycle dysregulation in BCBM provides a good basis for potential therapeutic investigation of cell cycle checkpoint kinase inhibitors in BCBM patients. Recent studies by Nguyen et al. (2019) further support the clinical relevance of our findings by demonstrating the activity of some CDK inhibitors against BM [32].

Through our integrative analysis across multiple datasets, we identified a core set of 34 consistently dysregulated genes in BCBM. Subsequently, we confirmed significant upregulation of 12 representative key genes in BM samples. The prognostic significance of these genes was demonstrated in survival analysis. All of the ten key genes analyzed showed significant association with poor outcomes in breast cancer patients. Importantly, eight of these genes (RRM2, CDCA8, CCNB1, LMNB2, FANCI, NCAPH, YWHAZ, and ESPL1) exhibited brain-specific over-expression when compared to other metastatic sites, further recommending their relevance in brain tropism.

The identification of FANCI as the gene with the strongest prognostic potential (HR = 1.78) and the most significant brain-specific expression is particularly noteworthy. FANCI is involved in DNA damage repair through the Fanconi anemia pathway, which is critical for maintaining genomic stability [33]. Its over-expression in BM suggests that enhanced DNA repair activity may be an important process for breast cancer cells to survive in the brain microenvironment. Similarly, the significant brain-specific over-expression of RRM2 involved in DNA synthesis, repair, and replication further strengthens the importance of maintaining genomic integrity in BCBM [34].

Although the neuronal gene NETO2 and DNA replication factor MCM2 did not show statistically significant brain-specific expression, their roles in BCBM might warrant further investigation. NETO2 has been implicated in neuronal function and its upregulation in BCBM might reflect adaptation to the neural environment, representing an interesting candidate for malignant formation. This finding is consistent with a previous study demonstrating the upregulation of neuronal-specific genes in BM that were not expressed in the primary tumor [35]. Park et al. show that metastatic cells adapt to the brain microenvironment by developing neuronal-like properties [35].

Among the hub genes with prior literature support, RRM2, CCNB1, ESPL1, MCM2, and YWHAZ have been individually associated with breast cancer progression and poor prognosis, though their brain-specific upregulation relative to other metastatic sites had not been formally established prior to this study. FANCI, which demonstrated the strongest prognostic effect (HR = 1.78) and most significant brain-specific upregulation, is a component of the Fanconi anemia DNA repair pathway; its specific involvement in BCBM has not been previously reported. For the remaining hub genes (CDCA8, LMNB2, NCAPH, SAC3D1, and FOXRED2), BM-specific literature is limited or absent, likely reflecting the historical focus of BCBM research on hypothesis-driven candidate-gene approaches rather than unbiased transcriptomic network analyses. The integrative approach employed in the current study addresses this gap and identifies these genes as novel candidates warranting further functional investigation.

Among the 12 validated hub genes, NETO2 and MCM2 warrant specific conceptual clarification. MCM2, a core component of the pre-replication complex and an established marker of cellular proliferative activity, is most appropriately interpreted as a pan-metastatic marker rather than a brain-selective one. Its upregulation across multiple metastatic sites reflects its fundamental role in sustaining proliferative demands of disseminated tumor cells regardless of target organ, and its lack of significant differential expression between brain and non-brain metastatic sites (*p* = 0.074) does not diminish its biological relevance. MCM2 may thus serve as a broadly applicable biomarker of metastatic aggressiveness, complementing the brain-specific markers identified in this study.

NETO2 presents a more complex interpretive challenge. Although it similarly did not reach significance in the brain-versus-other-sites comparison (*p* = 0.076), its trend toward higher expression in BM samples is notable given its neurobiological function. NETO2 encodes a transmembrane regulator of kainate-type ionotropic glutamate receptors and is predominantly expressed in neurons under physiological conditions. Its upregulation in BCBM therefore likely reflects context-specific transcriptional reprogramming facilitating tumor survival within the glutamate-rich neural microenvironment, rather than a non-specific proliferative response characteristic of pan-metastatic genes. We propose that NETO2 represents a neural microenvironment-responsive gene whose brain-specific association may reach statistical significance in larger, adequately powered cohorts. Functional studies examining its interaction with glutamatergic signaling pathways in BCBM models are warranted.

Our study has significant implications for the development of precision medicine strategies for BCBM. The identification of brain-specific genes provides potential biomarkers for predicting BM risk in breast cancer patients. For example, assessment of RRM2, CDCA8, and CCNB1 expression in primary tumors or circulating tumor cells might help identify patients at increased risk for BM. In addition, the consistent upregulation of these genes across multiple patient cohorts suggests their functional importance as therapeutic targets in BCBM. Furthermore, given the dominant role of cell cycle dysregulation, combinations of cell cycle inhibitors with agents targeting metabolic pathways or DNA repair regulation might be potentially effective against BCBM. The identified genes in our study could also serve as predictive biomarkers for response to specific therapies as part of personalized treatment.

### 4.2. Limitations and Future Directions

Despite our comprehensive analysis, several limitations need to be acknowledged. First, our study relies mainly on transcriptomic data, which may not fully reveal post-transcriptional or post-translational modifications that could affect protein function and cellular activities. Integration of proteomic and epigenetic data could provide a more complete understanding of the molecular mechanisms driving BCBM. Second, although we identified important associations between gene expression patterns and BM, these connections do not necessarily mean one causes the other. Both in vitro and in vivo studies are needed to confirm the functional roles of the key genes for brain metastatic progression identified in our study. Third, our analysis used bulk tissue samples, which include a mix of different cell types covering cancer cells, stromal cells, and immune cells. Higher-resolution techniques, such as single-cell sequencing, would provide better insights into the cellular heterogeneity within BM. This approach could also help elucidate the interactions between metastatic cancer cells and the brain microenvironment that facilitate brain colonization. Finally, our study was conducted using retrospective data from relatively small cohorts because surgically removed BM samples are rare. Prospective validation in larger patient cohorts would be valuable to confirm the clinical significance of our identified biomarkers and therapeutic targets. Furthermore, while each of the 12 identified hub genes was evaluated independently for its prognostic significance in the current study, their simultaneous dysregulation and extensive functional interconnections within the cell cycle regulatory axis suggest strong potential for integration into a combined multi-gene prognostic signature. The development and prospective validation of such a signature—for example, through multivariate Cox proportional hazards regression or machine learning-derived composite risk scoring—using individual patient-level gene expression data paired with clinical outcome data within a single well-powered cohort represents a key priority for future investigation. Such an approach has the potential to substantially enhance the clinical discriminatory power and translational applicability of these hub genes as prognostic biomarkers for BCBM.

## 5. Conclusions

Our comprehensive bioinformatic analysis has provided new insights into the molecular landscape of BCBM, highlighting cell cycle dysregulation, metabolic reprogramming, and immune microenvironment alterations as key factors. We have identified a set of key genes with prognostic significance and brain-specific expression patterns including RRM2, CDCA8, CCNB1, LMNB2, FANCI, NCAPH, YWHAZ, and ESPL1. These findings enhance our understanding of the molecular mechanisms driving brain metastasis in breast cancer and provide a foundation for the development of biomarkers for diagnostic, prognostic, and therapeutic approaches. Future functional studies focused on these key molecular alterations may lead to novel targeted therapies to improve outcomes for BCBM patients.

## Data Availability

The original contributions presented in this study are included in the article/Supplementary Material. Further inquiries can be directed to the corresponding author.

## Supplementary Materials

The following supporting information can be downloaded at https://www.mdpi.com/article/10.3390/diagnostics16081149/s1, Table S1: Data sources; Table S2: Complete list of 228 common differentially expressed genes (DEGs) upregulated in brain metastasis relative to primary breast tumors; Table S3: Summary of 12 validated hub genes associated with breast cancer brain metastasis.

## Abbreviations

The following abbreviations are used in this manuscript:

BM	Brain metastasis
BCBM	Breast cancer brain metastasis
GSEA	Gene set enrichment analysis
WGCNA	Weighted gene co-expression network analysis
DEGs	Differentially expressed genes
GEO	Gene expression omnibus
PPI	Protein–protein interaction
GO	Gene ontology
KEGG	Kyoto encyclopedia of genes and genomes
DMFS	Distant metastasis-free survival
HR	Hazard ratio
FDR	False discovery rate
NES	Normalized enrichment score
HER2	Human epidermal growth factor receptor 2
TNBC	Triple-negative breast cancer
BBB	Blood–brain barrier

## References

[1] Sung H., Ferlay J., Siegel R.L., Laversanne M., Soerjomataram I., Jemal A., Bray F. (2021). Global cancer statistics 2020: GLOBOCAN estimates of incidence and mortality worldwide for 36 cancers in 185 countries. CA Cancer J. Clin..

[2] Arnold M., Morgan E., Rumgay H., Mafra A., Singh D., Laversanne M., Vignat J., Gralow J.R., Cardoso F., Siesling S. (2022). Current and future burden of breast cancer: Global statistics for 2020 and 2040. Breast.

[3] Bray F., Laversanne M., Sung H., Ferlay J., Siegel R.L., Soerjomataram I., Jemal A. (2024). Global cancer statistics 2022: GLOBOCAN estimates of incidence and mortality worldwide for 36 cancers in 185 countries. CA Cancer J. Clin..

[4] Miller K.D., Nogueira L., Devasia T., Mariotto A.B., Yabroff K.R., Jemal A., Kramer J., Siegel R.L. (2022). Cancer treatment and survivorship statistics, 2022. CA Cancer J. Clin..

[5] Fares J., Fares M.Y., Khachfe H.H., Salhab H.A., Fares Y. (2020). Molecular principles of metastasis: A hallmark of cancer revisited. Signal Transduct. Target. Ther..

[6] Wang Y., Ye F., Liang Y., Yang Q. (2021). Breast cancer brain metastasis: Insight into molecular mechanisms and therapeutic strategies. Br. J. Cancer.

[7] Raghavendra A.S., Ibrahim N.K. (2024). Breast cancer brain metastasis: A comprehensive review. JCO Oncol. Pract..

[8] Lauko A., Rauf Y., Ahluwalia M.S. (2020). Medical management of brain metastases. Neurooncol. Adv..

[9] Niwińska A., Murawska M., Pogoda K. (2010). Breast cancer brain metastases: Differences in survival depending on biological subtype, RPA RTOG prognostic class and systemic treatment after whole-brain radiotherapy (WBRT). Ann. Oncol..

[10] Oehrlich N.E., Spineli L.M., Papendorf F., Park-Simon T.W. (2017). Clinical outcome of brain metastases differs significantly among breast cancer subtypes. Oncol. Lett..

[11] Tomasik B., Bieńkowski M., Górska Z., Gutowska K., Kumięga P., Jassem J., Duchnowska R. (2023). Molecular aspects of brain metastases in breast cancer. Cancer Treat. Rev..

[12] Sirkisoon S.R., Carpenter R.L., Rimkus T., Miller L., Metheny-Barlow L., Lo H.W. (2016). EGFR and HER2 signaling in breast cancer brain metastasis. Front. Biosci..

[13] Bos P.D., Zhang X.H.F., Nadal C., Shu W., Gomis R.R., Nguyen D.X., Minn A.J., van de Vijver M.J., Gerald W.L., Foekens J.A. (2009). Genes that mediate breast cancer metastasis to the brain. Nature.

[14] Miyamoto S., Yagi H., Yotsumoto F., Kawarabayashi T., Mekada E. (2006). Heparin-binding epidermal growth factor-like growth factor as a novel targeting molecule for cancer therapy. Cancer Sci..

[15] Lee B.C., Lee T.H., Avraham S., Avraham H.K. (2004). Involvement of the chemokine receptor CXCR4 and its ligand stromal cell-derived factor 1alpha in breast cancer cell migration through human brain microvascular endothelial cells. Mol. Cancer Res..

[16] Conrad C., Götte M., Schlomann U., Roessler M., Pagenstecher A., Anderson P., Preston J., Pruessmeyer J., Ludwig A., Li R. (2018). ADAM8 expression in breast cancer derived brain metastases: Functional implications on MMP-9 expression and transendothelial migration in breast cancer cells. Int. J. Cancer.

[17] Sevenich L., Bowman R.L., Mason S.D., Quail D.F., Rapaport F., Elie B.T., Brogi E., Brastianos P.K., Hahn W.C., Holsinger L.J. (2014). Analysis of tumour- and stroma-supplied proteolytic networks reveals a brain-metastasis-promoting role for cathepsin S. Nat. Cell Biol..

[18] Mustafa D.A.M., Pedrosa R.M.S.M., Smid M., van der Weiden M., de Weerd V., Nigg A.L., Berrevoets C., Zeneyedpour L., Priego N., Valiente M. (2018). T lymphocytes facilitate brain metastasis of breast cancer by inducing Guanylate-Binding Protein 1 expression. Acta Neuropathol..

[19] Avraham H.K., Jiang S., Fu Y., Nakshatri H., Ovadia H., Avraham S. (2014). Angiopoietin-2 mediates blood-brain barrier impairment and colonization of triple-negative breast cancer cells in brain. J. Pathol..

[20] Pavlova N.N., Zhu J., Thompson C.B. (2022). The hallmarks of cancer metabolism: Still emerging. Cell Metab..

[21] Cluntun A.A., Lukey M.J., Cerione R.A., Locasale J.W. (2017). Glutamine metabolism in cancer: Understanding the heterogeneity. Trends Cancer.

[22] Riecke K., Müller V., Neunhöffer T., Park-Simon T.-W., Weide R., Polasik A., Schmidt M., Puppe J., Mundhenke C., Lübbe K. (2023). Long-term survival of breast cancer patients with brain metastases: Subanalysis of the BMBC registry. ESMO Open.

[23] Lee J.Y., Park K., Lee E., Ahn T., Jung H.H., Lim S.H., Hong M., Do I.-G., Cho E.Y., Kim D.-H. (2016). Gene expression profiling of breast cancer brain metastasis. Sci. Rep..

[24] Iwamoto T., Niikura N., Ogiya R., Yasojima H., Watanabe K.-I., Kanbayashi C., Tsuneizumi M., Matsui A., Fujisawa T., Iwasa T. (2019). Distinct gene expression profiles between primary breast cancers and brain metastases from pair-matched samples. Sci. Rep..

[25] Zhang L., Ridgway L.D., Wetzel M.D., Ngo J., Yin W., Kumar D., Goodman J.C., Groves M.D., Marchetti D. (2013). The identification and characterization of breast cancer CTCs competent for brain metastasis. Sci. Transl. Med..

[26] Dong Y., Tu R., Liu H., Qing G. (2020). Regulation of cancer cell metabolism: Oncogenic MYC in the driver’s seat. Signal Transduct. Target. Ther..

[27] Tyagi A., Wu S.Y., Watabe K. (2022). Metabolism in the progression and metastasis of brain tumors. Cancer Lett..

[28] Schild T., Low V., Blenis J., Gomes A.P. (2018). Unique metabolic adaptations dictate distal organ-specific metastatic colonization. Cancer Cell.

[29] Berghoff A.S., Lassmann H., Preusser M., Höftberger R. (2013). Characterization of the inflammatory response to solid cancer metastases in the human brain. Clin. Exp. Metastasis.

[30] Ogiya R., Niikura N., Kumaki N., Yasojima H., Iwasa T., Kanbayashi C., Oshitanai R., Tsuneizumi M., Watanabe K.-I., Matsui A. (2017). Comparison of immune microenvironments between primary tumors and brain metastases in patients with breast cancer. Oncotarget.

[31] Lauko A., Thapa B., Venur V.A., Ahluwalia M.S. (2018). Management of brain metastases in the new era of checkpoint inhibition. Curr. Neurol. Neurosci. Rep..

[32] Nguyen L.V., Searle K., Jerzak K.J. (2019). Central nervous system-specific efficacy of CDK4/6 inhibitors in randomized controlled trials for metastatic breast cancer. Oncotarget.

[33] Shah R.B., Kernan J.L., van Hoogstraten A., Ando K., Li Y., Belcher A.L., Mininger I., Bussenault A.M., Raman R., Ramanagoudr-Bhojappa R. (2021). FANCI functions as a repair/apoptosis switch in response to DNA crosslinks. Dev. Cell.

[34] Zuo Z., Zhou Z., Chang Y., Liu Y., Shen Y., Li Q., Zhang L. (2022). Ribonucleotide reductase M2 (RRM2): Regulation, function and targeting strategy in human cancer. Genes. Dis..

[35] Park E.S., Kim S.J., Kim S.W., Yoon S.-L., Leem S.-H., Kim S.-B., Kim S.M., Park Y.-Y., Cheong J.-H., Woo H.G. (2011). Cross-species hybridization of microarrays for studying tumor transcriptome of brain metastasis. Proc. Natl. Acad. Sci. USA.

