# Supplementary material for: Integrative Bioinformatic Analysis Identifies Key Genes Driving Breast Cancer Brain Metastasis
Source: Diagnostics (Basel). 2026 Apr 13;16(8):1149. doi: 10.3390/diagnostics16081149 (PMC13115110; doi:10.3390/diagnostics16081149)
Supplement: Supplementary file 1 [file diagnostics-16-01149-s001.zip › diagnostics-4236034-supplementary.pdf]

Supplementary Table S1. Data sources.

Dataset	Website
1. GSE191230	https://www.ncbi.nlm.nih.gov/geo/query/acc.cgi?acc=GSE191230
2. GSE43837	https://www.ncbi.nlm.nih.gov/geo/query/acc.cgi?acc=GSE43837
3. GSE14018	https://www.ncbi.nlm.nih.gov/geo/query/acc.cgi?acc=GSE14018
4. GSE14017	https://www.ncbi.nlm.nih.gov/geo/query/acc.cgi?acc=GSE14017

Supplementary Table S2. Complete list of 228 common differentially expressed genes (DEGs) identified from GEO datasets GSE191230 and GSE43837.

All 228 DEGs are upregulated in brain metastasis (BM) relative to primary breast tumors. DEGs were identified by Venn diagram intersection of upregulated genes across both datasets.

No.	Gene Symbol
1	RFX4
2	AGT
3	ARFGEF3
4	DUSP9
5	SCG2
6	DIRAS2
7	SCRG1
8	MBP
9	MRPL45
10	SLC6A1
11	CDK12
12	RRM2
13	ADGRV1

14	SS18L1
15	LLGL2
16	DHCR24
17	FOXK2
18	DLG3
19	NETO2
20	PPP1R3D
21	PTK6
22	GSDMB
23	KRT18
24	TMED3
25	ELF3
26	XK
27	BAALC
28	YTHDF1
29	SIPA1L3
30	SLC35C1
31	PSMB3
32	TSEN54
33	NDUFA3
34	MRPS7
35	SOX9
36	NXT1
37	PPM1H
38	ITGA10
39	NARF

40	BEX2
41	IDI1
42	TSKU
43	CSTF1
44	PGK1
45	CSNK1D
46	FOXC2
47	TIGD2
48	NFS1
49	ACP2
50	ESPL1
51	ADGRG1
52	FRZB
53	APLN
54	MARCKSL1
55	S100A11
56	CDCA8
57	CCNB1
58	POLB
59	FAM174B
60	SPDEF
61	FBXL18
62	SHMT2
63	MRPL58
64	DDIT3
65	BSCL2

66	SRPRB
67	RECQL5
68	KAT6A
69	POP1
70	ACP1
71	ZBED4
72	PSRC1
73	PIK3R2
74	LSS
75	GSTA4
76	RPN2
77	AIFM1
78	GMDS
79	NDUFB9
80	GPM6B
81	SAC3D1
82	RABIF
83	PSMB2
84	FLVCR2
85	NCAPH
86	SPATA5L1
87	TPI1
88	SLC39A4
89	ZDHHC9
90	STX6
91	CD82

92	MCM2
93	ILF2
94	MRPS35
95	LRRC8B
96	NDRG1
97	TIMM8A
98	CLSTN1
99	UQCC2
100	NINL
101	FLAD1
102	SMUG1
103	LSM4
104	AHSA1
105	CRNKL1
106	ORMDL2
107	DNMT3A
108	DDB1
109	NAT9
110	CCDC6
111	LRPPRC
112	BCOR
113	HSPD1
114	PIGM
115	PDIA3
116	PRKDC
117	PPIA

118	YWHAZ
119	FANCI
120	ZNF512B
121	PSME4
122	VDAC1
123	CCDC120
124	MRPS11
125	LMNB2
126	DERL1
127	AKAP1
128	JMJD4
129	FAN1
130	AIMP2
131	NFKBIB
132	ZNF408
133	RPN1
134	NDUFA6
135	ABCB9
136	NVL
137	MRPS5
138	PRDX2
139	E2F6
140	KDELR2
141	ACOT7
142	JTB
143	TRAPPC3

144	NFYA
145	PSMB4
146	DIS3
147	ARF3
148	MAP2K3
149	NDUFB10
150	LONP1
151	UQCRFS1
152	NOLC1
153	TOMM22
154	CRELD2
155	KLHL12
156	PTDSS1
157	KIF1B
158	PPP2R2D
159	ZBTB39
160	MIA3
161	COMMD5
162	RAD21
163	SAR1B
164	EIF3I
165	DCLRE1A
166	C2CD2L
167	MPV17L2
168	PGAM1
169	PDCD6

170	C11orf24
171	CDK4
172	RPRD1B
173	CALR
174	AGO2
175	ENSA
176	APOOL
177	DHFR
178	COPZ1
179	NOP56
180	FAM50A
181	ELOVL1
182	UTP23
183	MCTS1
184	RER1
185	GOT2
186	FARSB
187	YIF1A
188	NAA50
189	HSPA4
190	WDR37
191	CCT7
192	FOXRED2
193	YTHDF2
194	ZFP41
195	ATP6V0C

196	ZNF250
197	DIDO1
198	NONO
199	RRBP1
200	COX6B1
201	PHF6
202	EMC7
203	COX19
204	SNRNP25
205	ARF1
206	TRPT1
207	CSPP1
208	PLAGL2
209	RPL8
210	MRPS26
211	PTK2
212	LDHA
213	PSMD2
214	GDI2
215	TIMM10
216	MRPL48
217	GET4
218	MAT2A
219	HSPA9
220	EIF2S1
221	SET

222	ZNF260
223	POLR1B
224	AREL1
225	CIAPIN1
226	SLC26A6
227	OSBP
228	XRCC6

Supplementary Table S3. Summary of 12 validated hub genes associated with breast cancer brain metastasis (BCBM).

No.	Gene Symbol	Full Gene Name	Functional Category	Key Biological Role in BCBM	Brain-Specific Over-expression†	Prognostic HR‡ (DMFS)
**1**	**RRM2**	Ribonucleotide Reductase Regulatory Subunit M2	Cell Cycle Regulation/DNA Synthesis	Catalyzes deoxyribonucleotide synthesis essential for DNA replication; promotes cell proliferation and metastatic potential	Yes ($p < 0.01$)	1.67
**2**	**NETO2**	Neuropilin and Tolloid-Like 2	Neuronal Adaptation	Modulates neuronal receptor function; upregulation in BCBM may reflect transcriptional adaptation to the brain microenvironment	Trend ($p = 0.076$)	1.53
**3**	**ESPL1**	Extra Spindle Pole Bodies Like 1 (Separase)	Cell Cycle Regulation	Protease essential for sister chromatid separation during mitosis; dysregulation promotes chromosomal instability	Yes ($p < 0.01$)	1.58
**4**	**CDCA8**	Cell Division Cycle Associated 8	Cell Cycle Regulation	Component of the chromosomal passenger complex; regulates mitotic spindle assembly checkpoint and cytokinesis	Yes ($p < 0.01$)	1.63
**5**	**CCNB1**	Cyclin B1	Cell Cycle Regulation	Master regulator of the G2/M cell cycle	Yes ($p < 0.01$)	1.47

				checkpoint; over-expression drives aberrant mitotic entry and metastatic proliferation		
**6**	**LMNB2**	Lamin B2	Chromosome Organization/Nuclear Structure	Structural component of the nuclear lamina; maintains nuclear integrity and regulates chromatin organization during cell division	Yes ($p < 0.01$)	1.29
**7**	**SAC3D1**	SAC3 Domain Containing 1	mRNA Export/Cell Cycle	Involved in mRNA export from the nucleus and cell cycle progression; role in BCBM is a novel finding of this study	N/A	N/A
**8**	**NCAPH**	Non-SMC Condensin I Complex Subunit H	Chromosome Organization	Subunit of the condensin I complex; essential for mitotic chromosome condensation and segregation fidelity	Yes ($p < 0.01$)	1.43
**9**	**MCM2**	Minichromosome Maintenance Complex Component 2	Cell Cycle Regulation/DNA Replication	Helicase component of the pre-replication complex; essential for DNA replication initiation and S-phase progression	Trend ($p = 0.074$)	1.52
**10**	**YWHAZ**	Tyrosine 3-Monooxygenase/Tryptophan 5-Monooxygenase Activation Protein Zeta	Cellular Signaling	14-3-3 family adaptor protein; modulates oncogenic signaling, chemotherapy resistance, and metastasis-promoting pathways	Yes ($p < 0.01$)	1.32
**11**	**FANCI**	FA Complementation Group I	DNA Damage Repair	Key component of the Fanconi anemia DNA repair pathway; over-expression may confer survival advantage under replicative stress in the brain microenvironment	Yes ($p < 0.001$)	1.78
**12**	**FOXRED2**	FAD-Dependent Oxidoreductase Domain Containing 2	Mitochondrial Metabolism	Involved in mitochondrial oxidative metabolism; novel association with BCBM identified in this study	N/A	N/A

Abbreviations: BCBM, breast cancer brain metastasis; DMFS, distant metastasis-free survival; FA, Fanconi anemia; FAD, flavin adenine dinucleotide; HR, hazard ratio; mRNA, messenger ribonucleic acid; N/A, not applicable; SMC, structural maintenance of chromosomes.

† Brain-specific over-expression was assessed by comparing expression levels in brain metastasis samples versus other metastatic sites (lung, liver, and bone) using Student's t-test; p-values are indicated accordingly. SAC3D1 and FOXRED2 did not reach statistical significance in this comparison and are therefore marked as N/A.

‡ Hazard ratios (HRs) for distant metastasis-free survival (DMFS) were derived from Kaplan–Meier Plotter analysis. SAC3D1 and FOXRED2 did not reach statistical significance in the survival analysis and are therefore marked as N/A. Refer to Figure 5 for complete Kaplan–Meier plots for the remaining ten hub genes.
